# A non-randomised pragmatic trial of a school-based group cognitive-behavioural programme for preventing depression in girls

**DOI:** 10.1080/22423982.2017.1396146

**Published:** 2017-11-07

**Authors:** Heléne Zetterström Dahlqvist, Evelina Landstedt, Ylva B Almqvist, Katja Gillander Gådin

**Affiliations:** ^a^ Department of Health Sciences, Mid Sweden University, Sundsvall, Sweden; ^b^ Department of Public Health and Clinical Medicine, Umeå University, Umeå, Sweden; ^c^ Centre for Health Equity Studies, Stockholm University, Stockholm, Sweden

**Keywords:** School, depressive symptoms, real-life setting, pragmatic trial, cognitive-behavioural, sexual harassment

## Abstract

The aim of this study was to investigate the effectiveness of the DISA-programme in preventing depressive symptoms (DS) in adolescent girls, as implemented in a real-world school setting, accounting for baseline socioeconomic and psychosocial factors, and to investigate whether the effects of these baseline variables on DS differed between intervention participants and non-participants. In this non-randomised pragmatic trial, an electronic questionnaire was disseminated in 2011 (baseline) and 2012 (follow-up) in schools in one municipality in northern Sweden. Pupils (total *n*=275; intervention participants identified in the questionnaire: *n*=53; non-participants: *n*=222) were 14–15 years old at baseline. The groups were compared by means of SEM. DISA could not predict differences in DS at follow-up in this real-life setting. In the overall sample, sexual harassment victimisation (SH) at baseline was associated with DS at follow-up and the estimate for SH increased in the DISA-participants compared to the overall sample.

## Introduction

A relatively common approach to prevent depression in adolescents has been to target the cognitive-behavioural aspects of adolescent coping strategies in the form of psychoeducational interventions in the school setting. This approach is based on cognitive vulnerability-stress interaction models of depression, which regard cognitive and behavioural characteristics of the individual as influencing the impact of adverse life events, and has been well established in adult populations [1–3]. In the developmental period of adolescence, however, the empirical and theoretical bases of this model are not as well confirmed [3]. Cole and Turner [4] propose a developmental hypothesis suggesting that negative cognitive styles are acquired in the transition from childhood to adolescence when children develop the capacity for, e.g., abstract reasoning. Hankin and Abela [5] have shown that it is possible to apply cognitive vulnerability-stress theories of depression in adolescence to predict depression in the transition from early to middle adolescence, as youth with high cognitive vulnerability who encountered more stressors were the most likely to exhibit increases in depressive symptoms. Abela and Hankin [6] have also shown that rumination moderates the association between the occurrence of stressors and the development of future depressive symptoms. In sum, even though the empirical and theoretical bases of cognitive vulnerability-stress interaction models in adolescents needs more development, there is some support that such models are applicable in youth.

Several reviews, however, have shown that there is a contradictive evidence base of the effectiveness of school-based preventive interventions targeting cognitive-behavioural vulnerability in youth. As reported by several reviews on the matter, some studies have been able to show an effect, while others have failed to do so [7–9]. One possible explanation for the negative results has been that many interventions were relatively brief, lasting only 8–20 hours, and may not have provided sufficient dosage [7]. There are, however, examples of relatively brief cognitive-behavioural interventions that have been shown to have an effect, e.g. LARS&LISA [10] and the Penn Resiliency Programme (PRP), the latter of which has been reported to be effective in reducing depressive symptoms as well as hopelessness [11].

However, evaluations of the PRP have also shown contradictory results, where investigators found the programme to be effective in two of the intervention schools, but not in a third school, and the authors could not determine the reason for this [12]. Most of these previous studies have been randomised and controlled trials, with researchers involved in assignment procedures and/or delivery (i.e. efficacy trials). Little is known about the effectiveness of this type of interventions when applied with less controls in community settings [13]. Drawing on Brown *et al*. [13], the current study focuses on the second step in the traditional research pipeline, by conducting an effectiveness trial embedded in the community without controls or supervision by researchers. Studies such as the current study are valuable in order to understand if effective treatments evaluated under controlled conditions remain effective when implemented naturalistically without such controls [14]. Further, the cognitive-behavioural programme that is the focus of this study, Depression in Swedish Adolescents (DISA), is already widely used in schools throughout Sweden [15] and parts of Finland [16], despite little knowledge of the effectiveness of the programme.

### Depression in Swedish Adolescents – DISA

DISA is based on the American Coping with Stress Course (CWS), which has been shown to be effective in selective interventions in people at elevated risk of depression and when delivered by trained mental health professionals (e.g. social workers and psychologists) [17]. DISA is a shortened Swedish version of the CWS and has been developed as a selective intervention by Clarke and Lewinsohn [18], together with the Centre for Public Health at the Stockholm County Council. To mirror Swedish conditions and the Swedish school curriculum, modifications and adjustments of the original manual and workbook have been made [15], such as replacing comic strips (personal communication, Annette Einar, January 2011). However, no study examining the DISA adaptation of CWS for this new cultural group has been published [19].

With the aim to “immunise” against future depression, the main objective of the DISA programme is to learn how to control irrational and negative thoughts, teach communication and problem-solving skills and how to create more pleasant events [18]. DISA consists of 10 group sessions of 1.5 hours each over 10 weeks, for a total dosage of 15 hours. Psychoeducational in nature and manual-based, the method was developed to be possible to undertake within school hours or as an extracurricular activity. In addition to the sessions, homework assignments are included as part of the training. The first session aims at giving an introduction of the 10-week programme. The following two sessions aim at giving an overview of depressive symptoms and the connection to stressful events. The remaining sessions focus on teaching the participants cognitive skills to identify and cope with irrational or negative self-perceptions and thought patterns, which are considered to be risk factors for the development and persistence of depressive symptoms [18]. (For a more in-depth description of the theoretical foundations of the DISA programme [20]. The current study is a follow-up of the Zetterström Dahlqvist et al. study. [20])

DISA groups are led by paraprofessionals such as teachers, school nurses, school counsellors or youth workers, which makes DISA similar to the beyondblue Schools Research Initiative [21] and the Penn Resiliency Programme [11]. In order to be able to lead DISA groups, the paraprofessionals take part in three days of theoretical training and have supervision during their first implemented group. Individual schools determine the time of the day that DISA is delivered, as well as teaching staff. Pupils are assigned to a DISA group based on each school’s assessments of whom they find appropriate for the intervention, on a first-come-first-served basis or via a general invitation to participate. In some cases, the choice of pupils reflects timetabling (personal communication, Eva-Mari Thomas, 3 September 2015).

#### Previous research about DISA

The programme is widely used in schools throughout Sweden as a universal intervention for both genders, but primarily as a selective intervention for girls only [15]. The effects on depressive symptoms of the DISA programme have only been evaluated scientifically in one study, with no control group [22]. In that study, DISA was implemented as a universal intervention to all pupils, girls as well as boys, in grade 8 (~14 years old) from a school located in an area with high mean income. Sixty-two eighth graders out of 68 agreed to participate and 55 pupils of both genders completed the questionnaire at follow-ups. Garmy *et al*. [22] report a reduction in mean and median scoring of depressive symptoms in girls at post-intervention and at 12-months follow-up, while in boys there was a decrease in symptoms at post-intervention only. However, as the authors point out, the lack of a control group in their study limits the conclusions that can be drawn regarding the effectiveness of the DISA programme. Furthermore, Garmy *et al*. [22] did not account for socioeconomic or psychosocial environmental factors. Also, the analyses were split by gender, which rendered low power, which further calls for caution regarding the results of their study.

In another study of DISA, we found that girls who participated in DISA had elevated depressive symptoms at baseline compared to girls not participating [20]. In addition, the pupils allocated to the DISA programme reported significantly higher median scorings on sexual harassment victimisation, personal relative affluence and less peer support. Furthermore, DISA participants reported borderline significantly higher levels of bullying victimisation [20]. This indicates that DISA, when implemented naturalistically by schools, was used as a targeted intervention rather than a selective one as it was originally intended [18]. As discussed previously, pupil assignment to a DISA group is usually determined based on each school’s assessments of whom they found to be appropriate for the intervention, on a first-come-first-served basis or via a general invitation to participate (personal communication, Eva-Mari Thomas, 3 September 2015). Unfortunately, no information is available regarding the schools’ rationale regarding which pupils were selected to participate in DISA.

### The present study

The pragmatic trial design of the current study enhances the understanding of the effectiveness of DISA in real-life conditions, but it also introduces methodological difficulties when it comes to confounding factors between intervention conditions. To address these confounding factors, the current study examines key environmental and individual circumstances that may influence the intervention. In order to enhance the understanding of the psychosocial circumstances under which mental health interventions are optimally effective, it is crucial to explore what characterises such circumstances [23], besides treating them as confounding factors. To the best of the authors’ knowledge, the current study is the first to examine the effectiveness of the DISA programme in preventing depressive symptoms in a pragmatic/real-world trial and is the first to investigate the role of socioeconomic *and* psychosocial environmental factors. Hence, the aims of the current study were two-fold; the first aim was to investigate the effectiveness of the DISA programme in preventing depressive symptoms in 14–15 year old girls, as implemented in a real-world school setting when taking into account baseline socioeconomic and psychosocial factors characterising circumstances in that setting. The second aim was to investigate whether the effects of the socioeconomic and psychosocial variables at baseline on depressive symptoms at follow-up differed between participants and non-participants in the DISA programme, i.e. does DISA moderate the effect of these variables?

## Methods

For the purposes of the present study, “real-world setting” means that the DISA programme was implemented by school staff in schools with no interference from the research team regarding assignment procedures or delivery of the programme. However, in order to learn more about the method, the first author has taken part in the three days theoretical training that all DISA group leaders undergo.

### Context

Data from female pupils from the Youth Health Development project (YHD project) was utilised for the purposes of the current study. As we have shown in a previous study [20], no schools in the municipality offered DISA to boys during 2011. The YHD project is a longitudinal study of health development in adolescents in the northern part of Sweden and is a part of a governmental investment involving six municipalities throughout Sweden. The overall aim of the YHD project was to find methods to promote mental health in adolescents, including development and implementation of such methods in school settings. Although the DISA programme had been implemented in and by schools in the geographical area of the YHD project prior to the start of the project, it was considered a potentially effective and worthwhile method to evaluate regarding effectiveness in a real-world setting within the YHD project framework. The municipality in which the YHD project longitudinal questionnaire was conducted is of medium size (~60,000 inhabitants) and is characterised by a diverse socioeconomic base, with a focus on tourism and small- and medium-sized enterprises.

In Sweden, most children begin their first year at school in the fall term when they reach the age of 7 and attendance is compulsory for all children up to the age of 16. Compulsory school is free of charge and may be either municipal or independent. The majority of schools are municipally run and most pupils attend a municipal school close to their home. Independent schools are run by corporations, foundations or associations, but are also free of charge [24].

## Design, procedure and participants

This study had a pragmatic trial design and was based on an electronic questionnaire that was administered in schools in the above-mentioned municipality twice over a 12-month follow-up period. The purpose of pragmatic trials is to evaluate the effectiveness of interventions in real-life routine practice conditions and generate results that can be generalised in routine practice settings. Since most results from exploratory trials, which aim at evaluating an intervention under optimal conditions, fail to be broadly generalisable, the pragmatic design has gained in popularity within the health sciences [25].

In the YHD project, all public and independent high schools with pupils in grades 7–9 were invited to participate. All public high schools (*n*=9) and one out of four independent schools agreed to participate. Intervention participants were identified in the follow-up questionnaire in which respondents were asked to indicate participation in DISA during the spring term of 2011. Data was collected in January 2011 for baseline (BL) and in January 2012 for follow-up (FU), which means that the time from post-intervention to FU is ~8 months. Informed consent was retrieved from pupils and parents. Pupils were informed about the aims of the questionnaire and that they could withdraw from participation at any time. The study was approved by the Umeå Regional Ethical Review Board (Dnr 09-179M). The electronic questionnaire was filled in on computers during school hours with a research assistant present.

**Figure 1.**
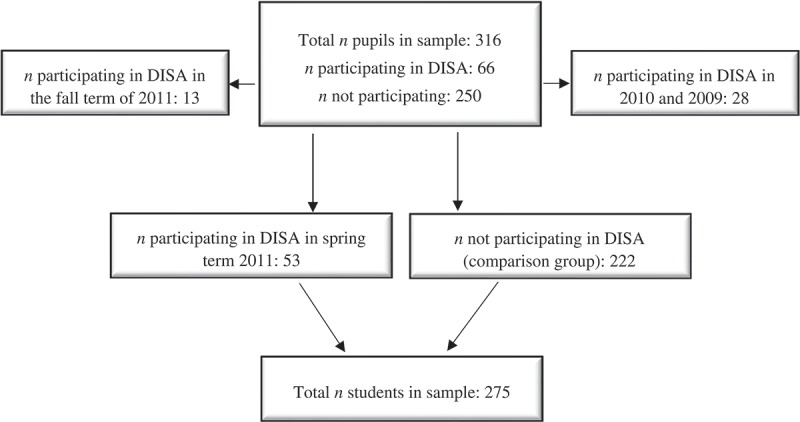
Respondents included in the analyses.

The response rate of the total sample (BL, *n*=1,482; FU, *n*=1,293), including both genders in grade 7–9, was 80.5% at BL and 79.5% at FU (girls, *n*=627 at BL; 559 at FU). The non-respondents differed between the schools by 10–40%, mainly due to differences in the school administrations’ engagement in providing adequate conditions to answer the questionnaire. Girls in grade 9 in 2011 (BL) were excluded from the analyses, since by 2012 (FU) they had been transferred to senior high school and were, thus, not eligible for follow-up. As some students leave compulsory school each year (after grade 9), only some of the students are the same from wave to wave. Of those who answered the questionnaire in 2011 (students in grades 7 and 8), 27.4% were lost to follow-up, although they were expected to answer the survey as they were still in compulsory school, i.e. in grade 8 or 9. As reported in Figure 1, the total sample in the present study was 316. Seven of the 10 schools included in the YHD project implemented at least one DISA group during 2011, rendering in total 66 girls who participated in DISA in 2011. However, in order not to inflate the results, 13 girls who participated in the DISA programme during the fall term of 2011 were excluded from the analyses because the time from post-intervention to the FU questionnaire was only ~2 months (Figure 1). Hence, only girls participating in DISA in the spring term of 2011 were included in the analyses (*n*=53), of which 7.5% (*n*=4) took part in DISA only a few times, reflecting low compliance. Twenty-eight girls participated in DISA in 2010 or in 2009 and were, thus, omitted from the analyses, rendering a corresponding number of intervention non-participants of 222, and they were, thus, assigned to a comparison condition. Twenty-eight per cent (*n*=15) of the intervention participants were in the same classroom as a non-participant (not shown in Figure 1).

### Measures

#### Outcome variable

The Centre for Epidemiologic Studies Depression Scale (CES-D) was used to measure levels of depressive symptoms [26,27]. It was developed for screening rather than for diagnostic purposes and was developed to identify current depression symptoms, with scores ranging from 0–60 and higher scores indicating more depressive symptoms [26–28].

#### Baseline characteristics variables

The Cronbach alpha of depressive symptoms (CES-D) at baseline was 0.89. One socioeconomic variable was personal relative affluence and respondents were asked to indicate how often (always, often, sometimes, rarely, never) in the past 6 months they have had enough money to be able to do the same things as their friends (scale range = 1–5, with lower scores indicating lower personal relative affluence). This variable has been shown to be associated with depressive symptoms in a larger sample from the YHD study. [29]. As the choice of baseline covariates was based upon a previous study of the same intervention [20], in order to be able to control for more possible confounders, the Family Affluence Scale (FAS) [30] was added to the models. Higher scores indicate higher family material affluence. Psychosocial variables were sexual harassment victimization, bullying victimisation and peer support. Respondents were asked about sexual harassment victimisation using an instrument previously used by Gruber and Fineran [31,32] and the AAUW study [33], by indicating how often they had experienced the following behaviours against their will during the past 6 months (never, once, a few times, many times): touched, grabbed or pinched you in a sexual manner; pushed you into a corner in a sexual manner; spread sexual rumours about you; commented on you, made a joke out of you or gesticulated in your direction in a sexual manner; looked at you in a manner that felt intrusive and sexual; shown or left you sexual images, photos or drawings; written sexual messages about you on bathroom walls or in locker rooms; called you a lesbian, fag or such words; pulled/pulled off your clothes in a sexual manner (e.g. pulled your bra straps, pulled your underwear or pulled up your skirt); tried to kiss or hug you; called you a “4 letter word”; commented on your looks, your body or your personal life in a sexual manner; spread comments about you or pictures of you on mobile phones; publicly commented on how attractive or unattractive you are. This scale has a range of 14–56, with higher scores indicating higher levels of sexual harassment victimisation, and demonstrates good internal consistency in the current sample (α=0.87). Bullying was measured by asking respondents to indicate if, in the past 6 months, it had happened that one or several pupils had teased, picked fights or shut the respondent out (no, never; yes, once; yes, a few times; yes, several times; yes, almost all the time, scale range = 1–5, higher scores indicating higher levels of victimisation). Peer support was measured by asking the following questions: “Does it happen that you are alone when you don’t want to?”; “Do you have as many friends you’d like?”; “Do you sometimes feel left out by your peer group?” (always; often; sometimes; rarely; never). Scale range = 0–12, with higher scores indicating more peer support. Cronbach’s alpha of this measure in the current sample was α=0.72.

#### Follow-up depressive symptoms

The CES-D had a Cronbach’s alpha of 0.77 at follow-up.

### Statistical analyses

#### Between-group differences at baseline

Between-group differences in mean scoring on depressive symptoms at baseline and follow-up, as well as baseline characteristics variables were calculated using independent samples *t*-tests. Between-group differences in grade level at baseline were analysed using Chi-squared statistics.

The proportion of missing data was as follows: CES-D 14.6% (BL), 11.4% (FU); peer support 4.7%; sexual harassment 5.4%; bullying 3.5%; personal relative affluence 0.9%; and FAS 0.6%. To handle missing items in the data, multiple imputation [34] was used in the independent sample *t*-tests calculations and the Chi-squared statistics. All between-group baseline differences analyses were conducted using SPSS 22.

#### Intervention effectiveness

**Figure 2.**
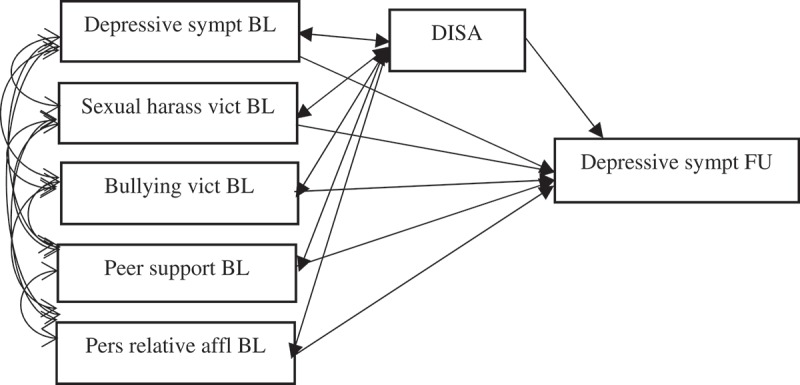
Step 1 of the analysis: the effect of the DISA programme on depressive symptoms at follow-up and direct effects of baseline covariates on depressive symptoms at follow-up. (Error terms have been omitted to avoid clutter.) Controlled for family affluence.

Besides the fact that 7.5% (*n*=4) of the girls took part in DISA only a few times, there was no information available on non-compliance regarding, e.g., homework assignments. Hence, all analyses are reported according to the intention-to-treat principle [35]. Intervention effectiveness was analysed by means of Structural Equation Modelling (SEM), using Mplus version 6.1. Structural equation models were estimated in two steps (*n*=275). The first step (see Figure 2) was to examine the effectiveness of the DISA programme on depressive symptoms at follow-up, while controlling for baseline differences in depressive symptoms. Hence, the change in depressive symptoms from baseline to follow-up was considered as a function of the intervention/no intervention. Such a regression-based approach corresponds at large to matched groups. In addition, the model included direct effects from all study variables at baseline (i.e. depressive symptoms, sexual harassment victimisation, bullying, poor peer support and lower personal relative affluence) on depressive symptoms at follow-up, as well as correlations between all baseline variables. Hence, we treat them as traditional confounding variables that are adjusted for in the analysis of the association between DISA and depressive symptoms. The FAS scale was added as a control variable. At an initial stage of the analysis, grade was also adjusted for, but, due to poor model fit, the decision was made to exclude it. It should, however, be noted that the adjustment for grade altered the point estimates to a very limited extent.

**Figure 3.**
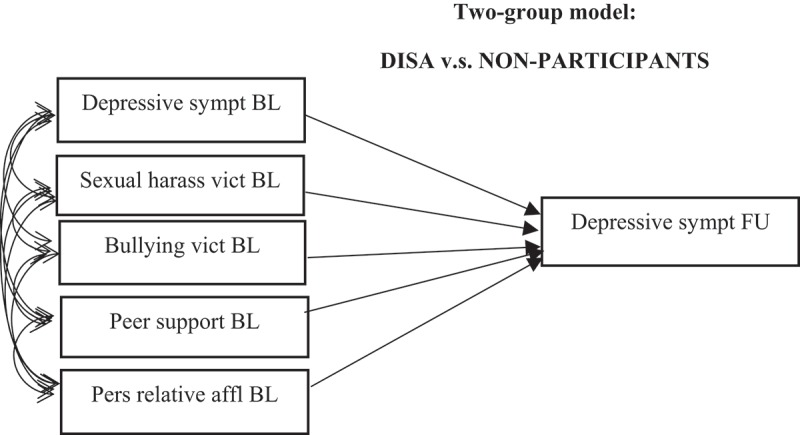
Step 2 of the analysis; two-group comparisons: differences between DISA participants and non-participants in terms of the effect of baseline covariates on depressive symptoms at follow-up. (Error terms have been omitted to avoid clutter.) Controlled for family affluence.

The second step (see Figure 3) was to study whether the effects of the study variables at baseline on depressive symptoms at follow-up differed between participants and non-participants in the DISA programme, adding a multiple group comparison to the SEM model. Hence, in this second step, DISA is considered the moderating variable. Here, as well, the model encompassed correlations between all variables at baseline. This second model was also controlled for family affluence.

Missing data was handled using Full Information Maximum Likelihood (FIML) estimation. Furthermore, since individuals attending the same school class may be expected to be more similar to each other compared to individuals attending different classes [36], the models were estimated with cluster robust standard errors.

The estimated models were assessed using a combination of several fit indices: the Root Mean Square Error of Approximation (RMSEA), which preferably should be at or below 0.05, although cut-offs up to 0.10 are also acceptable [37]; the Comparative Fit Index (CFI) and the Tucker-Lewis Index (TLI), which both should optimally be at or above 0.95 [37,38], although cut-offs at or above 0.90 are considered acceptable [39]; and the Standardised Root Mean Square Residual (SRMR), which should be at or below 0.08 [38]. All Alpha levels were set to 95%.

## Results

### Baseline characteristics

Table 1 reports baseline differences between the intervention group and the non-participants. Girls participating in DISA had significantly higher scores on depressive symptoms and significantly lower scores on personal relative affluence compared to their non-participating peers. In addition, girls participating in DISA reported more bullying victimisation than the non-participants. There were no baseline mean differences in sexual harassment victimisation, peer support or family affluence (FAS). Also shown in Table 1, at the 1-year follow-up, mean scoring on depressive symptoms in the comparison group had increased to approximately the same level as the intervention group, who had not changed their mean scoring. The change in the comparison group was statistically significant: baseline, Mn=14.45, SD=9.19, follow-up, Mn=17.14, SD=6.65, *t*=4.593, df=221, *p*<0.001 (not shown in Table 1).

**Table 1.** Inter-group differences at pre-intervention and at 1-year follow-up by intervention condition.

Independent samples t-test							
Variable	Intervention group (*n*=53), mean (SD)	Comparison group (*n*=222), mean (SD)	*p*-value*^a^*	*t*	Mean difference	SE of mean difference	95% CI of mean difference
Depressive symptoms BL	19.14 (9.83)	14.45 (9.13)	0.001	−3.295	−4.690	1.423	−7.49– −1.90
Depressive symptoms FU	18.65 (6.73)	17.14 (8.03)	0.160	1.407	−1.506	1.071	−3.60– 0.59
Sexual harassment BL	4.24 (5.58)	2.87 (4.60)	0.074	1.790	−1.364	0.762	−2.86– 0.13
Bullying BL	1.92 (1.10)	1.61 (0.97)	0.042	−2.032	−0.315	0.155	−0.62– –0.01
Peer support BL	15.74 (3.21)	16.23 (2.90)	0.294	1.050	0.487	0.463	−0.42–1.40
Personal relative affluence BL	3.87 (1.11)	4.38 (0.83)	0.003	3.137	0.509	0.162	0.19–0.83
FAS BL	5.69 (1.18)	5.58 (1.10)	0.559	0.584	0.104	0.178	−0.25–0.45
χ^2^-test							
	Intervention group (*n*=53), % (*n*)	Comparison group (*n*=222), % (*n*)					
	Proportion % (*n*)		χ^2^	*p*-value			
Grade level 7 T1	54.8 (121)	47.2 (25)	0.987	0.359			

*^a^* Significance based on a two-tailed test. Differences are significant with *p*<0.05.

### Intervention effectiveness

The model fit indices for step 1 of the analysis were RMSEA: 0.052; CFI: 0.930; TLI: 0.848, SRMR: 0.034, whereas the following model fit statistics were produced for step 2: RMSEA: 0.066; CFI: 0.090; TLI: 0.799; SRMR: 0.058. Thus, while both models show acceptable values for RMSEA, CFI and SRMR, the TLI value is lower than optimal. For both steps 1 and 2 of the analysis, the estimated models fitted data better than the empty models/baseline models.

The path coefficients from the first step of the SEM analysis are presented in Table 2. First of all, the results show no effect of the DISA programme on depressive symptoms at follow-up, while there was a statistically significant auto-regressive path from depressive symptoms at baseline to depressive symptoms at follow-up (0.255, *p*=0.002). The rather moderate size of the estimate of depressive symptoms at baseline in this sample suggests that there was a predictive power of depressive symptoms over a 1-year follow-up period, although not a very strong one. Moreover, there was a positive, statistically significant and somewhat stronger effect of sexual harassment victimisation at baseline on depressive symptoms at follow-up (0.307; *p*≤0.001). Bullying, peer support and low personal relative affluence, on the other hand, did not predict any significant changes in depressive symptoms over time.

**Table 2.** The effectiveness of the DISA programme on depressive symptoms at follow-up. Standardised path coefficients from structural equation modelling (*n*=275).

	Estimate*^a^*	95% *p*-value*^b^*
DISA	0.005	0.931
*Baseline variables*		
Depressive symptoms	0.255	0.002
Sexual harassments	0.307	<0.001
Bullying	−0.095	0.157
Peer support	−0.095	0.253
Low personal relative affluence	−0.029	0.584

*^a^* Controlled for family affluence. *^b^* Significance based on a two-tailed test. Differences are significant with *p*<0.05.

Table 3 presents the standardised path coefficients from the second step of the analysis where, in contrast to the previous set of results, the analysis is grouped by DISA participation. In other words, the results show whether the effects of depressive symptoms, sexual harassment and bullying victimisation, peer support and low personal relative affluence at baseline on depressive symptoms at follow-up differed between DISA participants and non-participants.

**Table 3.** Differences between DISA participants and non-participants in terms of the effect of study variables at baseline on depressive symptoms at follow-up. Standardised path coefficients from structural equation modelling (*n*=275).

Baseline variables	Estimate*^a^*	*p*-value*^b^*
*DISA participants* (*n*=53)		
Depressive symptoms	0.383	0.036
Sexual harassment victimisation	0.565	<0.001
Bullying	0.136	0.591
Peer support	0.002	0.993
Low personal relative affluence	0.078	0.266
*Non-participants* (*n*=250)		
Depressive symptoms	0.234	0.018
Sexual harassment victimisation	0.266	0.001
Bullying	−0.118	0.104
Peer support	−0.138	0.062
Low personal relative affluence	−0.048	0.437

*^a^* Controlled for family affluence. *^b^* Significance based on a two-tailed test. Differences are significant with *p*<0.05.

Among the DISA participants, the effects of depressive symptoms and sexual harassment victimisation at baseline were statistically significant and of medium strength (0.383, *p*=0.036.and 0.565, *p*≤0.001, respectively), suggesting that those who have been exposed to this kind of harassment were more likely to experience an increase in depressive symptoms over time.

Among the non-participants, there was a statistically significant path from depressive symptoms at baseline to follow-up (0.234, *p*=0.018). Moreover, among the non-participants, the measure of sexual harassment victimisation at baseline was also significantly associated with depressive symptoms at follow-up (0.266, *p*=0.001), albeit weaker than in the group of DISA participants. This suggests that, also in the non-participant group, those who report sexual harassment victimisation are more likely to have increased depressive symptoms over time. Neither peer support nor bullying and low personal relative affluence were significantly associated with depressive symptoms at follow-up in either group.

## Discussion

This non-randomised, pragmatic effectiveness study focused on investigating the effectiveness of the DISA programme on depressive symptoms in a real world setting at an 8-month follow-up. Contrary to previous research on the DISA programme [22] and its American forerunner, the CWS [17], as well as to that of other trials of cognitive-behavioural programmes [10,11,40], we could not show that DISA had an effect on depressive symptoms. This may be due to the fact that, in the current sample, intervention participants had elevated levels of depressive symptoms (> 16) [27] at baseline which the pupils in, for example, Garmy *et al*. [22] had not. Also, in their study, all pupils in eighth grade were invited to participate, i.e. DISA was implemented as a universal intervention [22]. In the current study, girls with elevated symptoms in grades 7 and 8 had taken part in DISA, i.e. DISA was implemented as a targeted intervention [20].

In the overall sample, sexual harassment victimisation at baseline was associated with depressive symptoms at follow-up and was of medium strength. When we separated the analyses according to intervention condition (Step 2), the estimate of sexual harassment victimisation for the DISA participants *increased* considerably compared to the direct effects of sexual harassment victimisation in the total sample (Step 1). As there were no significant differences in mean scoring of sexual harassment victimisation between the groups at baseline, this difference in strength of association between the DISA participants and the non-participants cannot be explained by differences in exposure. Hence, participating in DISA seems to inflate the effects of sexual harassment victimisation, which seems to contradict a cognitive vulnerability-stress interaction model of depression. This is an important and unexpected finding. Kvist Lindholm and Zetterkvist Nelson [41] have shown in a qualitative study of girls’ experiences of participating in DISA that, in groups that do not have a “safe” atmosphere, DISA could actually be a trigger of already ongoing destructive interactions such as bullying and harassment. The authors claim that this result is explained by the self-disclosure component in DISA where participants are expected to disclose personal feelings and thoughts (in general, not necessarily about sexual harassment victimisation experiences) as part of the learning process. With that said, there was no information available about if pupils participating in DISA considered their group as safe or not. It is also possible that there was an actual difference in scoring of sexual harassment victimisation between the groups at baseline that could not be detected due to low power. This is supported by the fact that, in a previous study, we found that there was a difference in *median* scoring in basically the same sample (13 girls were omitted in the current study) [20]. Nevertheless, agreeing with a cognitive vulnerability-stress interaction model of depression, Allen and Sheeber [42] discuss how the increased complexity and stressfulness of the contexts in which young people find themselves can outrun cognitive as well as emotional maturation and, thus, overwhelm their competencies. Allen and Sheeber [40] stress the importance to acknowledge how the rapid changes in both social and biological trajectories during the transition from childhood to adolescence may create a discordance between social demands and maturational capacities. Sexual harassment can be viewed as an extreme form of negative social demand and DISA does not seem to be able to alter the negative effects of victimisation.

Zetterström et al. have shown that sexual harassment victimisation is stable over time in girls; if one is victimised in grade 7, one is also victimised in grades 8 and 9 and it would be reasonable to argue that sexual harassment victimisation is a considerable stressor over time for these victimised girls lives [43]. Furthermore, other scholars have shown that sexual harassment at school is part of everyday life [44–46] and that sexual harassment victimisation is associated with poor mental health outcomes [29,31,32]. Hence, preventing sexual harassment victimisation seems to be important as a factor to consider when planning interventions to support girls’ positive mental health development. The developers of the DISA method claim that DISA is able to “immunise” against future depressive symptoms [18]. However, in the face of sexual harassment victimisation, DISA does not seem to be able to do just that. An interpretation would be that learning to reverse one’s negative thoughts in the face of elevated depressive symptoms *and* sexual harassment victimisation is not a beneficial way to promote a healthy development in these girls.

### Methodological considerations

In the current study, the response rate and respondents not lost to follow-up of the total sample was satisfying. Internal consistency was also satisfactory in the multiple items instruments used. All data is based on self-reports and memory bias may be present because DISA participants were identified in a questionnaire rather than in school records. Few respondents reported participation in DISA during the spring of 2011, which rendered relatively low power. Model fits were acceptable regarding RMSEA, CFI and SRMR, but less so regarding TLI [34,37,39]. Due to the study’s design, another methodological issue was the risk of contamination, since 28% of the intervention participants had non-participants in the same classroom. By employing cluster robust standard errors on the class level in the SEM-models, this could be dealt with in a satisfactory way. Due to the observed baseline mean differences between the groups, inter-group comparisons are problematic and, at an early stage of the analyses, we matched the two groups using propensity score techniques. However, this rendered even lower power. Hence, we chose to proceed with the analyses without matched groups. Instead, baseline differences in depressive symptoms were controlled for in Step 1 of the analysis. Therefore, we analyse the change in depressive symptoms from baseline to follow-up as a function of the DISA participation/no participation. Such a regression-based approach corresponds at large to matched groups. Furthermore, by conducting the second step of the analyses – the multi-group model – standardised parameter estimates for each group were provided for each studied variable. By doing so, group differences in the studied variables were accounted for, something which is commonly overlooked in whole-sample data, such as paired *t*-tests or analysis of variance [47].

In effect trials of cognitive-behavioural interventions there are issues that should be addressed. First, we lack information about group leaders’ experience and skills or to what extent they stayed true to the manual. Second, the girls selected to participate in DISA had elevated levels of depressive symptoms, i.e. a score >16, which is the threshold of relevance for clinical depression [27], and DISA was never intended for that group [18]. DISA may possibly have a different effect if used strictly as a selective intervention. However, the results of the current pragmatic trial should be able to be generalised to other municipalities in Sweden, given that intervention participants have the same CES-D scoring at BL (≥16). The results should, however, not be generalised to other cognitive-behavioural interventions, since the theoretical underpinnings of DISA have not been tested in this study.

The current study is unique so far as having investigated a school-based cognitive-behavioural programme with a pragmatic/real-world setting design and it has proven itself by showing that, when schools did not deliver the DISA programme the way they were meant to, it was not possible to show effectiveness. In addition, including baseline circumstances has shown that sexual harassment victimisation in school is an important factor to consider when understanding results from studies of cognitive-behavioural interventions. Furthermore, only evaluating with randomised controlled trials (RCT) may bias the evidence base of an intervention’s effectiveness [14] and pragmatic/real-world trials should be considered complimentary to RCTs [48].

### Conclusions and implications for further research and practice

DISA could not predict differences in depressive symptoms at follow-up in a sample of girls with elevated depressive symptoms. When planning interventions to support girls’ positive mental health development, preventing sexual harassment victimisation seems to be an important factor, and, arguably, has a greater potential to prevent depressive symptoms in girls than the DISA programme.

As discussed in the background section in Gillham *et al*.’s [12] study, effects of the Penn Resiliency Programme were found in two schools, but not in a third. In light of the results in the current study regarding sexual harassment victimisation, the psychosocial situation in the third school could have been “hostile” as far as bullying and harassment are concerned. We suggest that, in future evaluations of cognitive-behavioural programmes in schools, these factors should be taken into consideration.

Despite the obvious challenges with pragmatic/real-world designs, it is important not to refrain from evaluating interventions with such designs [14] and the results of this study should be considered to be complimentary to any future RCTs. We know of no reason why the current municipality would be unique in regards to assigning girls with elevated depressive symptoms to the DISA programme and these results can help schools review and, if needed, revise their assignment process to the DISA programme. With that said, if schools are to continue their use of DISA, the programme should also be evaluated in a large scale randomised controlled trial. Nevertheless, DISA has been the object of extensive critique [49,50] and, arguably, future research should also engage in developing ways to prevent sexual harassment in school and, by so doing, it is likely that they have a better chance to prevent the onset of depressive symptoms.
